# DEPRESSION, INFLAMMATION, AND THE MODERATING ROLE OF METFORMIN: RESULTS FROM THE MIDUS STUDY

**DOI:** 10.1093/geroni/igac059.1639

**Published:** 2022-12-20

**Authors:** Sumaiyah Syed, Iris Yang, Stephanie Wilson

**Affiliations:** Southern Methodist University, Dallas, Texas, United States; Southern Methodist University, Dallas, Texas, United States; Southern Methodist University, DALLAS, Texas, United States

## Abstract

There is a well-established link between depression and aging-related inflammation, where depression can spur inflammation and affect physical health. Previous animal trials have shown compelling evidence that the use of metformin, the first line of treatment for diabetes, can offset inflammation. These findings have not yet been extended to human samples. This study examined the association between depression and inflammation, specifically the moderating role of metformin usage in middle-aged and older adults. We predicted that metformin would attenuate the link between depression and inflammation. Participants in Project 4 of the Midlife in the United States (MIDUS) Study (n=1255, Mage = 57) provided data on medication use, depressive symptoms, and inflammatory cytokines interleukin (IL)-6, tumor necrosis factor (TNF)-α, and C-reactive protein (CRP). Controlling for age, sex, body mass index (BMI), and comorbidity burden, metformin use moderated the association of depressive symptoms with IL-6 and CRP, but not TNF-α. Higher levels of depressive symptoms were significantly associated with higher interleukin 6 (IL-6) (p <.001) and C-reactive protein (CRP) (p = .033) among those not using metformin. However, this effect was not significant among those using metformin, suggesting that metformin usage may attenuate the relationship between depression and inflammation. These findings support the potential of metformin in mitigating the link between depression, a well-known behavioral risk factor, and inflammation, a key source of biological aging. Metformin, an accessible and commonly prescribed drug, may have a hidden anti-aging role that may protect against the inflammatory effects of depression.

